# SnowRemovalNet: BEV-Guided 3D Point Feature Learning for LiDAR De-Snowing in Autonomous Driving

**DOI:** 10.3390/s26206604

**Published:** 2026-10-18

**Authors:** Jinhyeok Choi, Dukjo Kong, Jiwoong Kim

**Affiliations:** 1 Purpose Built Mobility Group, Korea Institute of Industrial Technology (KITECH), Buk-gu, Gwangju 61012, Republic of Korea; j2nhyeok@kitech.re.kr; 2 Graduate School of AI Policy and Strategy, Gwangju Institute of Science and Technology (GIST), Buk-gu, Gwangju 61005, Republic of Korea

**Keywords:** autonomous driving, adverse weather, LiDAR, point cloud denoising, curriculum learning

## Abstract

In heavy snowfall, LiDAR point clouds contain numerous noise points caused by scattering and reflection from snow particles. In particular, snow and non-snow points are often spatially intermingled, making fine-grained pointwise discrimination challenging. However, existing LiDAR de-snowing methods have not sufficiently focused on this problem. Therefore, this study proposes SnowRemovalNet, a supervised LiDAR de-snowing network designed for fine-grained pointwise discrimination in complex snowy scenes. The proposed method incorporates global contextual information extracted from a bird’s-eye view (BEV) representation into 3D point feature learning. This enables the network to jointly consider the spatial distribution of snow noise and the local geometric relationships of individual points. To account for label uncertainty near the boundaries between snow and non-snow regions, the snow-noise likelihood of each point is represented as a continuous probability between 0 and 1. This value serves as the probabilistic label during training. On the CADC and WADS datasets, the proposed model effectively distinguishes spatially intermingled snow and non-snow points. Using only 30 training frames per dataset, the model achieves F1-scores of 98.10% on CADC and 97.23% on WADS. The processing time is 17–59 ms per frame.

## 1. Introduction

**Figure 1:**
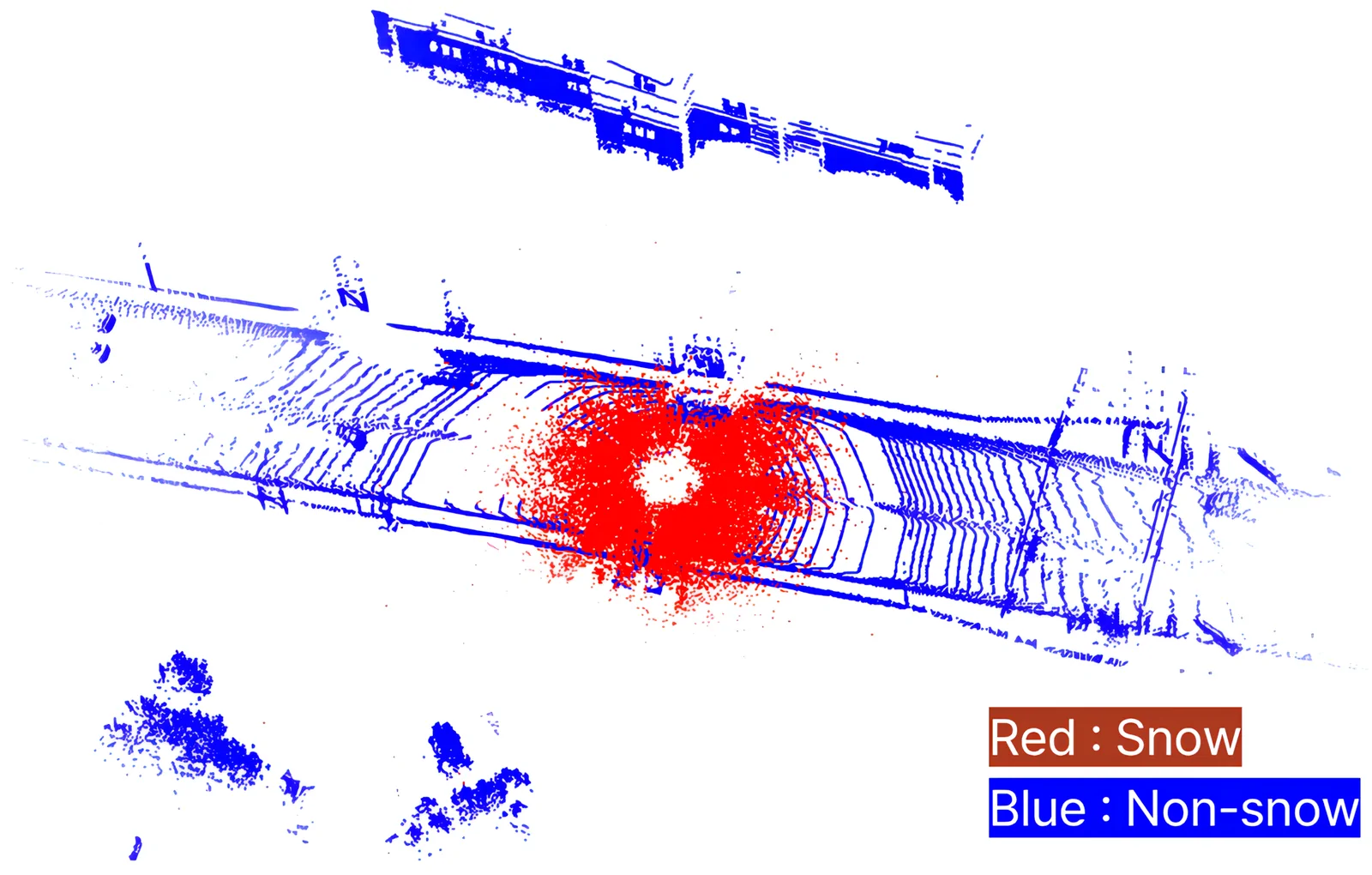
LiDAR point cloud in a heavy snowfall environment. Red and blue points indicate snow and non-snow points, respectively.

LiDAR point clouds are crucial for perception and localization in autonomous driving. However, heavy snowfall, as shown in Figure 1, generates numerous snow noise points, significantly degrading perception and localization performance. Statistical filtering methods based on distance and reflectance intensity have been proposed [1,2,3,4,5,6,7,8]; however, they have shown limited accuracy and real-time performance in extreme snowfall environments. Recently, deep learning-based methods have been proposed to handle complex snow patterns; however, two main limitations remain.

First, projecting LiDAR point clouds onto 2D representations inevitably loses detailed three-dimensional geometric information.

Second, the binary labeling strategy commonly used in supervised approaches cannot effectively model the uncertainty of ambiguous points. Assigning each point a label of either 0 or 1 can lose or distort boundary-region information, which may degrade discriminative performance and lead to inaccurate classification boundaries.

To address these limitations, this study proposes a deep learning-based LiDAR de-snowing network that leverages 2D contextual information from bird’s-eye view (BEV) images to enhance 3D point feature learning. Specifically, it extracts 3D local features based on Dynamic Graph CNN (DGCNN) [9] and modulates them with BEV-derived contextual features. Additionally, continuous probabilistic labels replace binary labels to capture pointwise uncertainty during training. The proposed method also employs curriculum learning [10], first focusing on highly confident points and progressively incorporating more ambiguous data, and an uncertainty-weighted loss function for stable and uncertainty-aware training.

The main contributions of this study are as follows:

1. A BEV-guided Edge Conv Block that modulates 3D point features with the global contextual features extracted from the BEV images.
2. Probabilistic-label-based training without manual annotation, in which the soft labels of our previous study [11] set the curriculum stages and loss weights.
3. A lightweight network with 35,925 parameters, trained with 30 frames per dataset, that processes one frame in 17 ms on CADC and 59 ms on WADS.

## 2. Related Work

LiDAR de-snowing methods are broadly divided into statistical filters and learning-based methods. Most learning-based methods share the two limitations noted in Section 1. First, a projected 2D representation, such as a range image, loses part of the 3D geometric relationships between neighboring points. Second, hard labels cannot express the uncertainty of spatially intermingled snow and non-snow points.

Statistical filters exploit the sparser distribution of snow-induced points relative to points on object surfaces. DROR [2] removes a point when the number of its neighbors within a search radius falls below a threshold. DSOR [1] removes a point when its mean distance to its *k* nearest neighbors exceeds a threshold. Both filters scale the radius or threshold with the distance from the sensor. Because snow-induced points also tend to exhibit low reflectance intensity [5], most other filters apply such a neighborhood test only to low-intensity snow candidates. LIOR [5], the adaptive two-stage filter [7], AGDOR [4], and the neighbor-intensity-based filter [8] apply radius-based outlier removal to the candidates. In contrast, DDIOR [3] and LIDSOR [6] apply DSOR-based filtering.

ASDR (Adaptive Snow-candidate and DSOR-based snow Removal) [11], proposed in our previous study, determines the intensity range of snow candidates for each frame from the intensity distribution of points near the sensor. It then applies DSOR to these candidates. The same study converts the ASDR output into soft labels refined by C-GLP (Confident Seed-based Graph Label Propagation); these labels are used here to train the proposed network. DCOR [12] applies the radius-based test within each laser channel with a search radius that increases with the distance from the sensor.

Learning-based methods differ mainly in how supervision is obtained. WeatherNet [13] performs supervised CNN-based pointwise segmentation on cylindrical range images and was designed primarily for rain and fog. AdverseNet [14] removes rain, snow, and fog noise with a single supervised network based on a cylindrical tri-perspective view representation. LiSnowNet [15] and SLiDE [16] do not require ground-truth labels. LiSnowNet separates snow in range images by combining a CNN with sparse representations from the Fourier and wavelet transforms. SLiDE identifies snow in a self-supervised manner by the difficulty of reconstructing the depth along each LiDAR ray. However, the sparsity prior can fail to separate snow from object structures with similar patterns. Reconstruction difficulty can also be high at object boundaries, sparse structures, and distant points.

Other studies extend the problem beyond a single frame or a single task. The multiframe denoising framework in [17] registers three consecutive frames and applies a time outlier removal filter. The 4DenoiseNet [18] framework combines *k*-nearest-neighbor convolutions within the current scan and toward the previous scan. The spatio-temporal conditional random field method in [19] propagates snow confidence from high-certainty points to uncertain ones and can be extended to sequential scans. The robust multi-task learning network in [20] shares a PointNet encoder among denoising, single-object segmentation, and completion branches.

Unlike these studies, the proposed method performs de-snowing as a single task on a single frame without registration between consecutive frames, making it complementary to multiframe approaches that exploit temporal consistency. It addresses the two limitations above by learning features directly from 3D points, with BEV images used only as contextual guidance, and by training with probabilistic labels that express pointwise uncertainty.

## 3. Snow Noise Characteristics and Representations

Due to the scattering and absorption of laser beams by snow particles, snow-induced points tend to exhibit lower reflectance intensity than typical non-snow points [5]. Furthermore, in heavy snowfall, snow particles closer to the sensor along the LiDAR beam path may generate returns before the laser reaches the actual target. This results in numerous noise points in the near-field region.

**Figure 2:**
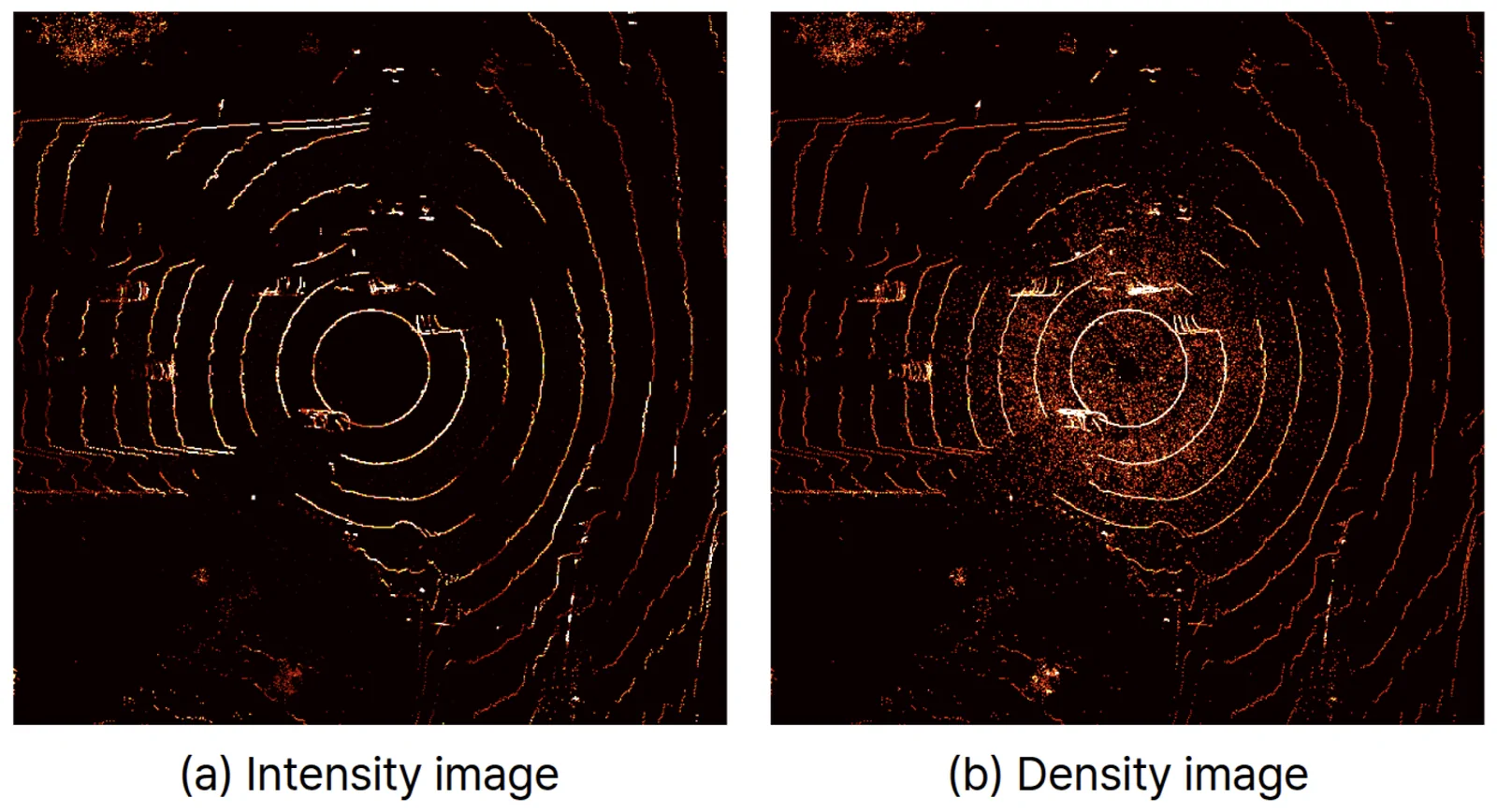
BEV images for a single-frame LiDAR scan. (**a**) Intensity image representing the average reflectance intensity of the points in each grid cell. (**b**) Density image representing the number of points in each grid cell.

Figure 2 shows BEV images. The intensity image in Figure 2a highlights the low reflectance intensity of snow-induced points, while the density image in Figure 2b reveals the concentration of noise points in the near-field region. These observations indicate that BEV images effectively capture the global distribution of snow noise.

However, the BEV image projects 3D point clouds onto a 2D plane, partially losing vertical information and detailed 3D geometric relationships between points. In contrast, the 3D point representation preserves local geometric relationships but is limited in capturing the global distribution of snow noise in heavy snowfall. Therefore, this study incorporates 2D global contextual information from the BEV intensity and density images into 3D point feature learning.

## 4. Method

### 4.1. Network Architecture Overview

**Figure 3:**
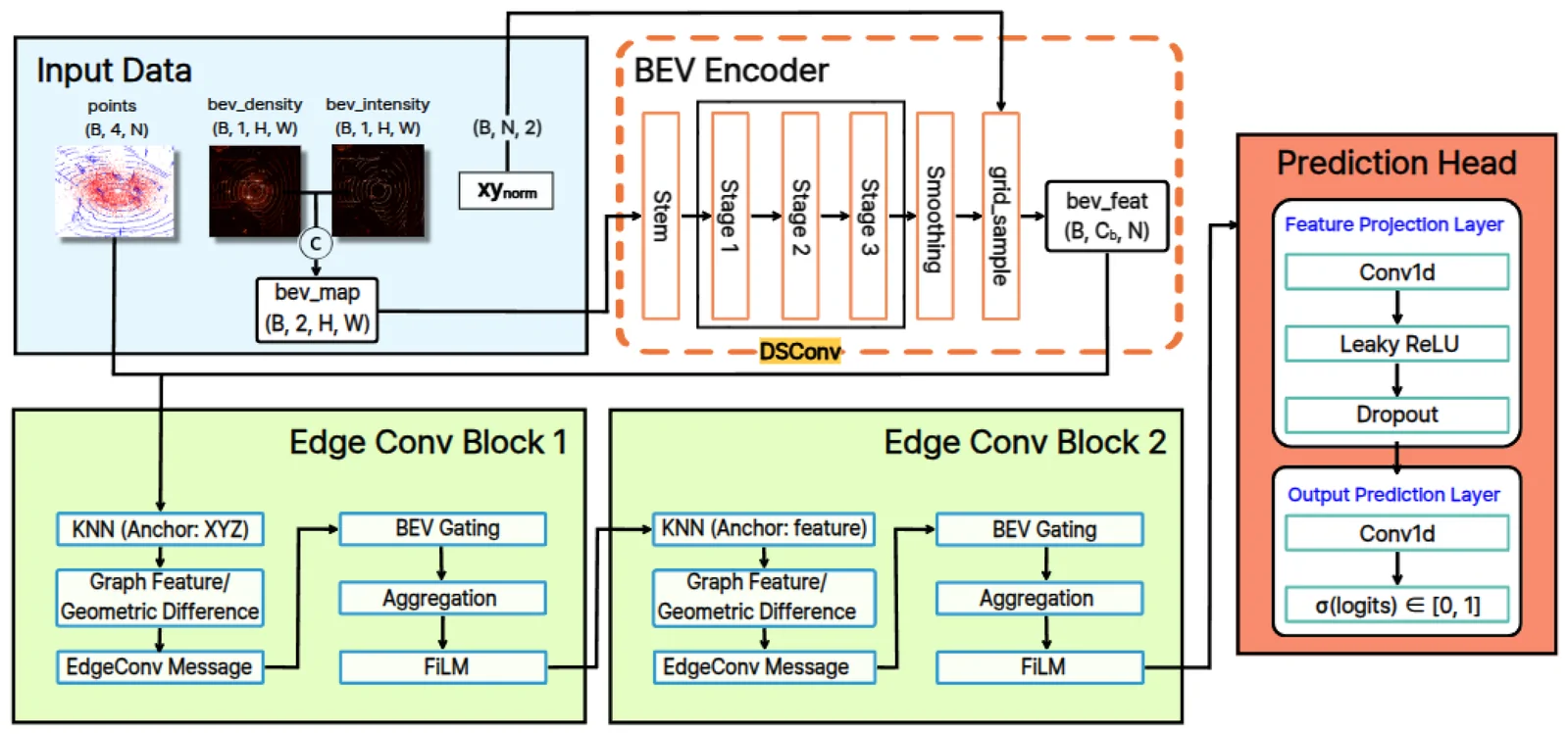
Overall architecture of SnowRemovalNet. The colors distinguish the input data, BEV Encoder, Edge Conv Blocks, and Prediction Head, and the circled C denotes channel-wise concatenation.

As shown in Figure 3, SnowRemovalNet consists of a BEV Encoder, Edge Conv Blocks, and a Prediction Head.

The BEV Encoder extracts 2D contextual features representing the spatial distribution of snow noise from the intensity and density images and samples them at each 3D point position. The Edge Conv Blocks learn local geometric features from the 3D neighbor relationships between points, using the BEV features as guidance. Finally, the Prediction Head outputs the probability of snow noise for each point.

### 4.2. Probabilistic Label Generation and Utilization

Publicly available snowy LiDAR datasets do not provide dense pointwise labels that represent the continuous likelihood of snow noise, and manual annotations are typically binary labels. Such binary labels may be insufficient to represent the uncertainty in ambiguous regions where snow and non-snow points are spatially intermingled.

Therefore, this study uses probabilistic labels that represent the snow-noise likelihood of each point as a continuous value in [0,1]. These labels are generated offline by applying the ASDR filter and C-GLP of our previous study [11]. ASDR generates binary pseudo-labels through adaptive snow-candidate generation and DSOR-based filtering. These pseudo-labels are then converted into soft labels and refined by C-GLP using neighboring point information. In contrast, the network architecture and the training scheme that uses label uncertainty are newly proposed in this study, and only SnowRemovalNet is used at inference.

Unlike binary labels, these probabilistic labels have values close to 0 for clearly non-snow points, close to 1 for clearly snow points, and close to 0.5 for ambiguous boundary points. This allows the model to learn decision boundaries that reflect the inherent uncertainty in the data.

The probabilistic labels are also utilized in curriculum learning [10] and loss function design. A curriculum mask $M_{i}\in \left\{0,1\right\}$ is defined for each point *i*, and only the selected points are considered in the loss calculation.

The basic classification loss for each point is defined by the Binary Cross Entropy (BCE) loss in (1). Here, $y_{i}$ is the probabilistic label, $x_{i}$ is the logit predicted by the network, and σ(·) denotes the sigmoid function.

(1) $${\mathrm{BCE}}_{i}=-\left[y_{i}\log\left(\sigma (x_{i})\right)+\left(1-y_{i}\right)\log\left(1-\sigma (x_{i})\right)\right].$$

To include the reliability of the probabilistic label in the loss function, the uncertainty weight $w_{uncertain,i}$ is defined as in (2).

(2) $$w_{uncertain,i}=\max\left(0.5,2\cdot \left|y_{i}-0.5\right|\right).$$

The final loss function is constructed by combining the BCE loss, uncertainty weight, and curriculum mask as shown in (3).

(3) $$L=\frac{\sum_{i=1}^{N}\left(M_{i}\cdot w_{uncertain,i}\cdot {\mathrm{BCE}}_{i}\right)}{\sum_{i=1}^{N}\left(M_{i}\cdot w_{uncertain,i}\right)}.$$

The curriculum mask $M_{i}$ follows a three-stage step schedule over the training epochs e=1,…,E with the stage boundaries $t_{1}=\left\lceil E/3\right\rceil$ and $t_{2}=\left\lceil 2E/3\right\rceil$, as defined in (4).

(4) $$M_{i}\left(e\right)=\left\{\begin{array}{cc} 1, & \mathrm{if}\;e\leq t_{1}\;\mathrm{and}\;\left(y_{i}\leq 0.1\;\mathrm{or}\;y_{i}\geq 0.9\right),\\ 1, & \mathrm{if}\;t_{1}<e\leq t_{2}\;\mathrm{and}\;\left(y_{i}\leq 0.3\;\mathrm{or}\;y_{i}\geq 0.7\right),\\ 1, & \mathrm{if}\;e>t_{2},\\ 0, & \mathrm{otherwise}. \end{array}\right.$$

With E=100, the three stages cover epochs 1–34, 35–67, and 68–100. The curriculum training procedure is summarized in Algorithm 1.
**Algorithm 1** Curriculum training of SnowRemovalNet**Input:** Training frames $D_{tr}$ and validation frames $D_{val}$ with probabilistic labels $y_{i}$; number of epochs *E*
**Output:** Final network parameters $\theta^{*}$
  1:$t_{1}\leftarrow \left\lceil E/3\right\rceil$, $t_{2}\leftarrow \left\lceil 2E/3\right\rceil$, $L_{val}^{*}\leftarrow \infty$  2:**for** e=1 **to** *E* **do**  3:      Stage s←1 if $e\leq t_{1}$; s←2 if $t_{1}<e\leq t_{2}$; otherwise s←3  4:      **for** each mini-batch of training samples drawn from $D_{tr}$ **do**  5:            Compute $M_{i}\left(e\right)$ of every point for stage *s* as in (4)  6:            Compute ${\mathrm{BCE}}_{i}$ and $w_{uncertain,i}$ as in (1) and (2)  7:            Compute L as in (3) and update θ with Adam  8:      $L_{val}\leftarrow$ mean of $w_{uncertain,i}\cdot {\mathrm{BCE}}_{i}$ on $D_{val}$, without the mask  9:      **if** s=3 and $L_{val}<L_{val}^{*}$ **then**10:            $L_{val}^{*}\leftarrow L_{val}$, $\theta^{*}\leftarrow \theta$11:**return** $\theta^{*}$


### 4.3. Input

The proposed method uses the following inputs.

#### 4.3.1. 3D Point Cloud

The 3D point cloud is the primary input to the model, and each point includes 3D coordinates (x,y,z) and reflectance intensity information. The shape of the tensor is (B×4×N), where *B* is the batch size, 4 is the number of channels, and *N* is the number of points.

#### 4.3.2. 2D BEV Map

The 2D BEV map is obtained by projecting the 3D point cloud onto a 2D grid in the XY plane. In this study, a BEV image denotes a single-channel 2D representation that encodes one type of point-cloud attribute on the BEV grid, whereas a BEV map denotes a multi-channel tensor constructed by concatenating multiple BEV images along the channel dimension.

As shown in Figure 2, the model generates an intensity image and a density image. Each image is constructed as a tensor of shape (B×1×H×W), where *H* and *W* denote the height and width of the BEV grid, respectively. The BEV grid covers ±30 m in *X* and *Y* with a cell size of 0.1 m, so that H=W=600. The two images are concatenated along the channel axis, resulting in a single BEV map of size (B×2×H×W), which is then passed to the BEV Encoder.

#### 4.3.3. Normalized 2D Coordinates ($xy_{norm}$)

This tensor stores the mapping between each 3D point and its projected location in the BEV map. It has a shape of (B×N×2), and each point contains the normalized *x* and *y* coordinates, scaled to the range of [−1,1].

### 4.4. BEV Encoder

**Figure 4:**
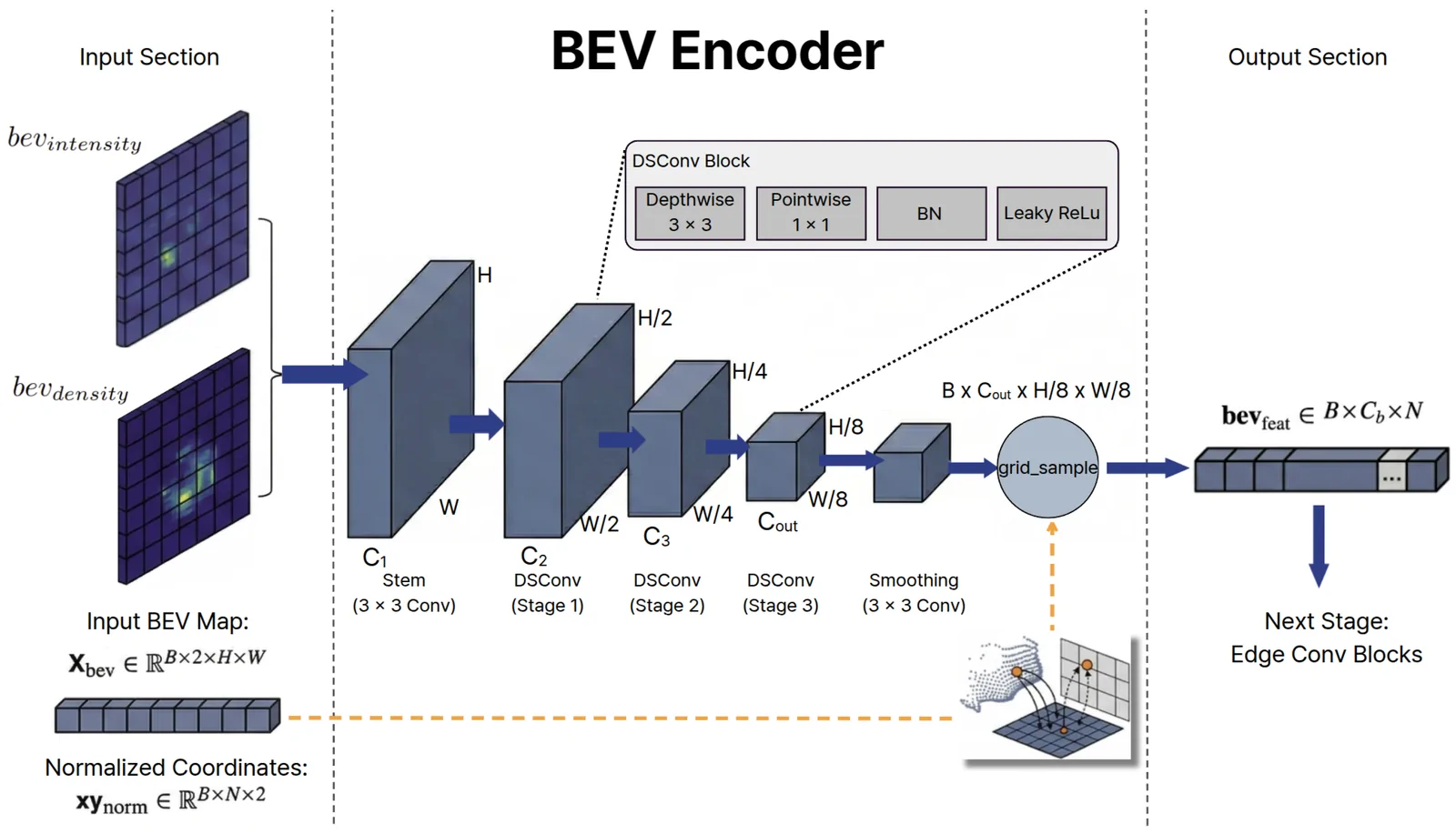
BEV Encoder structure. Blue arrows indicate the feature flow, and the orange dashed line indicates the normalized 2D coordinates of the points passed to grid sampling. The dotted lines show the internal structure of a DSConv block, and the vertical dashed lines separate the input, encoder, and output sections.

The BEV Encoder efficiently compresses the spatial contextual information of the 2D map through Depthwise Separable Convolution (DSConv) [21] and extracts the feature vector ($bev_{feat}$) corresponding to each 3D point. The detailed structure of the module is shown in Figure 4.

#### 4.4.1. Stem Layer

The Stem Layer applies a 3×3 convolution to the 2-channel input BEV map ($X_{bev}\in R^{B\times 2\times H\times W}$) consisting of $bev_{intensity}$ and $bev_{density}$. In this process, the number of channels is expanded to $C_{1}$, and the information from both images is combined to generate the initial BEV features.

#### 4.4.2. Depthwise Separable Convolution (DSConv)

The feature map from the Stem Layer passes through three DSConv blocks for feature extraction and dimensionality reduction. DSConv separates the operations into depthwise convolution and pointwise convolution to improve computational efficiency. At each stage, the resolution is halved. The number of channels is expanded from $C_{1}=16$ to $C_{2}=32$ and $C_{3}=64$ in Stages 1 and 2. In Stage 3, the number of channels is reduced to $C_{out}=16$. As a result, a BEV feature map with a resolution of H/8×W/8 (75×75) and $C_{out}$ channels is generated.

#### 4.4.3. Smoothing Layer

The Smoothing Layer applied after DSConv strengthens the spatial consistency of the feature map and suppresses noise through a 3×3 convolution. Batch normalization (BN) and LeakyReLU then produce the output with $C_{out}$ channels.

#### 4.4.4. Grid Sampling

Finally, the features corresponding to each point are sampled based on the normalized 2D coordinates ($xy_{norm}$) of each 3D point. Using the PyTorch (version 2.11.0) grid_sample function, point-specific features are extracted from a feature map of size $\left(B\times C_{out}\times H/8\times W/8\right)$. The final output is represented as $bev_{feat}\in R^{B\times C_{b}\times N}$, where $C_{b}=C_{out}$.

### 4.5. Edge Conv Block

3D point feature extraction is performed based on the Edge Conv Block of DGCNN [9]. The proposed module also incorporates 2D contextual information from the BEV Encoder to enhance the point representation for snow noise discrimination.

#### 4.5.1. KNN-Based Neighbor Selection

First, the neighbors of each point are searched for using KNN (*k*-Nearest Neighbors). Each block applies different criteria depending on the information extraction stage.

- **Edge Conv Block 1 (XYZ-based KNN):** Since meaningful point features are not yet available at the initial stage, KNN with k=16 is performed in the original XYZ coordinate space.
- **Edge Conv Block 2 (Feature-based KNN):** After the first Edge Conv Block, each point is represented by learned features that encode local geometric characteristics. Therefore, KNN with k=12 is performed in the learned feature space rather than in the original XYZ space. The KNN distances are computed on a fixed random 16-dimensional projection of the 64-dimensional point features.

#### 4.5.2. Graph Feature/Geometric Difference

The core of the Edge Conv Block is to combine graph features and geometric differences to compute the relationships between points.

- **Graph Feature:** The feature difference between the center point *i* and its neighbor *j* is computed as in (5).
  (5) $$f_{ij}^{graph}=f_{j}-f_{i}.$$
  The final edge feature $e_{ij}$ concatenates $f_{ij}^{graph}$ with the feature of the center point ($f_{i}$), as shown in (6).
  (6) $$e_{ij}=\mathrm{concat}\left(f_{ij}^{graph},f_{i}\right).$$
- **Geometric Difference:** The positional difference between points ($\Delta p_{ij}$) in (7) reflects the spatial structure and provides distance and directional information.
  (7) $$\Delta p_{ij}=xyz_{j}-xyz_{i}.$$

#### 4.5.3. Message Passing

The message $m_{ij}$ is generated by combining the edge feature $e_{ij}$ and the geometric difference $\Delta p_{ij}$ as shown in (8). ϕ is a neural network that includes a 1×1 convolution and batch normalization, which efficiently extracts the relationships between points.

(8) $$m_{ij}=\phi \left(e_{ij},\Delta p_{ij}\right).$$

#### 4.5.4. BEV-Based Gating

Using the $bev_{feat}$ generated by the BEV Encoder, weights are assigned to the messages. As shown in (9), the gate $g_{ij}$ is calculated by combining the BEV information of point *i* and its neighbor *j*.

(9) $$g_{ij}=\sigma \left(g_{bev}\left(\left[bev_{feat,i},bev_{feat,j}\right]\right)+h_{geo}\left(\Delta p_{ij}\right)\right).$$

Here, σ is the sigmoid function, and $g_{bev}$ and $h_{geo}$ are neural networks that process the concatenated BEV features and the geometric difference, respectively. The final message with the gate applied is as shown in (10).

(10) $$m_{ij}^{\prime }=g_{ij}⊙m_{ij}.$$

#### 4.5.5. Aggregation

Max pooling is used in the aggregation process. Let the neighbor set of point *i* be denoted as N(i), and the updated feature $f_{i}^{\prime }$ is as shown in (11).

(11) $$f_{i}^{\prime }=\max_{j\in N(i)}m_{ij}^{\prime }.$$

#### 4.5.6. FiLM (Feature-Wise Linear Modulation) [22]

The 3D local feature $f_{i}^{\prime }$ is modulated using the global contextual information from the 2D BEV. As shown in (12), the neural network ψ takes $bev_{feat,i}$ as input and predicts the scale ($\gamma_{i}$) and bias ($\beta_{i}$).

(12) $$\left[\gamma_{i},\beta_{i}\right]=\psi \left(bev_{feat,i}\right).$$

For training stability, $\gamma_{i}$ is transformed into $\gamma_{i}^{\prime }$, which is restricted to the range of [0.5,1.5] through (13).

(13) $$\gamma_{i}^{\prime }=1.0+0.5\cdot \tanh\left(\gamma_{i}\right).$$

The final output $f_{out}$ is calculated through an affine transformation as shown in (14) and passed to the next layer after batch normalization.

(14) $$f_{out}=\gamma_{i}^{\prime }⊙f_{i}^{\prime }+\beta_{i}.$$

### 4.6. Prediction Head

The Prediction Head takes the point features $f_{out}$ as input and predicts the snow noise probability for each point. This module consists of the Feature Projection Layer and the Output Prediction Layer.

The Feature Projection Layer consists of a 1×1 convolution with 64 output channels, LeakyReLU, and dropout. It refines the point representation into a form suitable for snow noise discrimination.

The Output Prediction Layer transforms each point feature into a single scalar logit $x_{i}$ through a 1×1 convolution. Then, a sigmoid function is applied to compute the snow noise probability ${\hat{y}}_{i}\in \left[0,1\right]$ for each point. During the inference stage, points with ${\hat{y}}_{i}\geq 0.5$ are classified as snow noise. The final output is a pointwise snow noise probability tensor of shape (B×1×N).

## 5. Experiments

### 5.1. Experimental Setup

Three public datasets were used: CADC [23], WADS [24], and Boreas [25], collected using a 32-channel Velodyne VLP-32, a 64-channel LiDAR, and a 128-channel Velodyne Alpha Prime LiDAR sensor, respectively.

CADC mainly contains snowy urban scenes, where snow-induced points are often intermingled with valid points from vehicles, traffic signs, and buildings. In contrast, WADS includes snowy rural and open-road scenes, where snow noise often overlaps with roadside objects and natural terrain. Some Boreas scenes contain irregular vegetation and tree-like structures, which can further complicate the distinction between snow and non-snow points.

Since CADC does not provide pointwise snow labels, all quantitative metrics on CADC were computed against manual annotations of the test data, which were not included in the training data. They were labeled point by point by inspecting only the raw point cloud, without referring to the ASDR and C-GLP outputs. Points floating in the air near the sensor and isolated points without neighboring points at long range were labeled as snow, yielding 7378 snow points (15.65%) out of 47,132. The pointwise labels provided by WADS were also manually re-annotated with the same criteria. Boreas also lacks pointwise snow labels; therefore, its snow-removal results were evaluated quantitatively in terms of LiDAR odometry accuracy in Section 5.4.2.

To assess generalizability across different sensors and datasets, the model trained on WADS was also applied to CADC and Boreas without retraining.

Table 1 lists the implementation details of SnowRemovalNet. For each dataset, 30 frames with probabilistic labels were randomly split into 21 training frames and 9 validation frames. Each training sample consisted of $N_{s}$ points randomly drawn anew every epoch from the points within 30 m of one training frame, because most snow points are located within 30 m of the sensor. Incomplete mini-batches were discarded.

All experiments were conducted on a system with an AMD Ryzen 9 9950X3D CPU, 64 GB RAM, and an NVIDIA GeForce RTX 5080 16 GB GPU. The evaluation metrics are defined as follows:

- **Learning Time (s, ↓):** The total time required to train the network. Traditional statistics-based methods are excluded because they do not require training.
- **Processing Time (ms, ↓):** The time required to process one frame of the point cloud. Processing times exceeding 100 ms may be insufficient for real-time operation.
- **Precision (%, ↑):** The ratio of points predicted as snow noise that are actually snow noise.
- **Recall (%, ↑):** The ratio of actual snow noise points that are successfully removed by the model.
- **F1-score (%, ↑):** The harmonic mean of Precision and Recall.

### 5.2. In-Dataset Evaluation

In this section, the model was trained and evaluated on the same dataset to analyze the snow-removal performance under each sensor and scene condition.

#### 5.2.1. Comparison with Existing Methods

**Figure 5:**
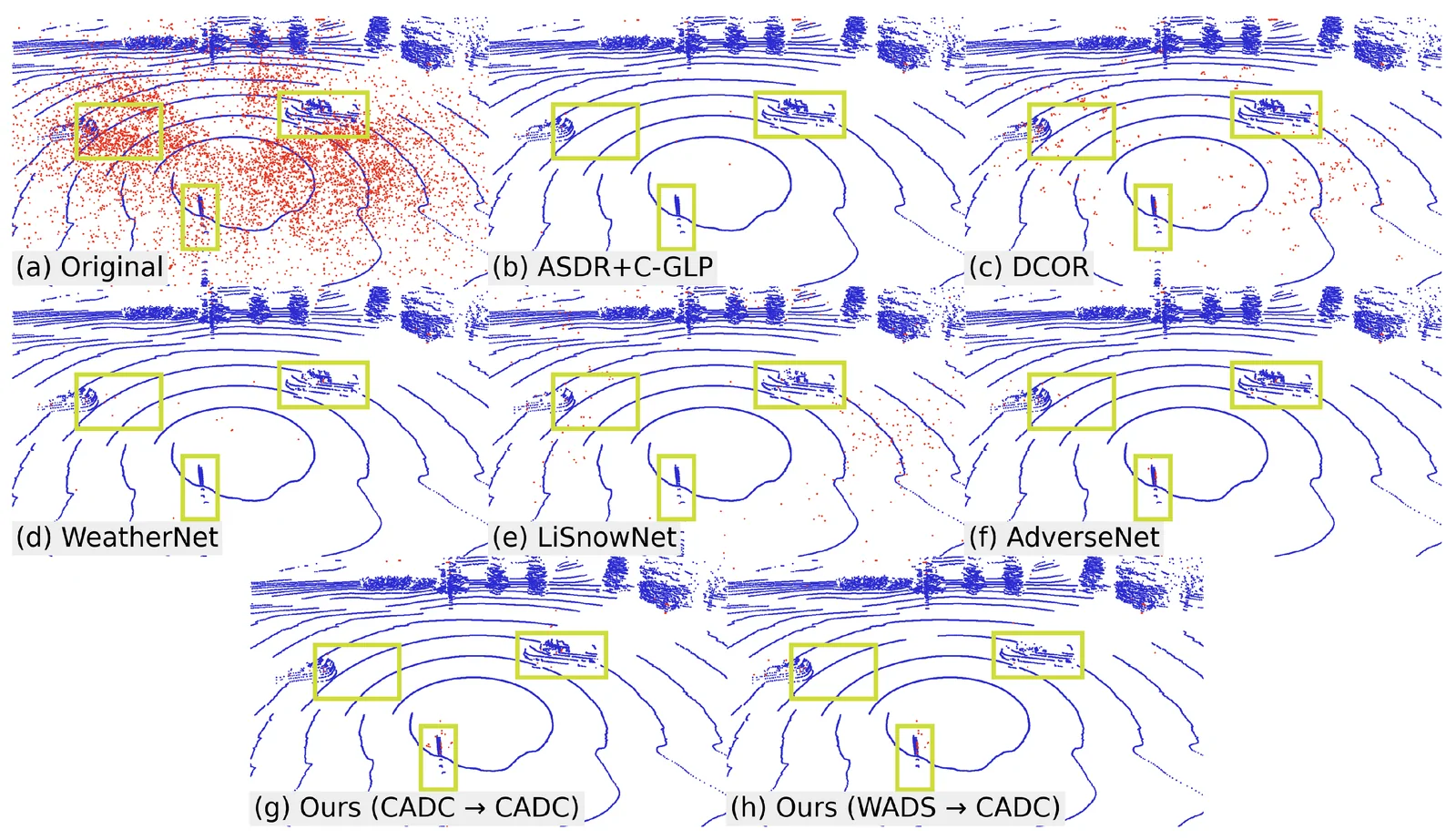
Qualitative comparison of snow-removal results on the CADC dataset. (**a**) Original point cloud. (**b**) ASDR+C-GLP labels. (**c**) DCOR. (**d**) WeatherNet. (**e**) LiSnowNet. (**f**) AdverseNet. (**g**) SnowRemovalNet trained and tested on CADC. (**h**) SnowRemovalNet trained on WADS and tested on CADC. Red and blue points indicate snow and non-snow points, respectively, and the yellow boxes highlight the regions around nearby vehicles and traffic signs.

On CADC, as shown in Table 2, the proposed method achieved a Precision of 98.09%, Recall of 98.10%, and F1-score of 98.10%. This F1-score was the highest among the compared de-snowing methods, excluding the offline ASDR+C-GLP labels. As shown in Figure 5g, the proposed method effectively removed snow noise around the vehicle while stably preserving nearby vehicles and traffic signs. In contrast, LiSnowNet tended to leave residual snow noise near the traffic signs or remove more valid object points.

**Figure 6:**
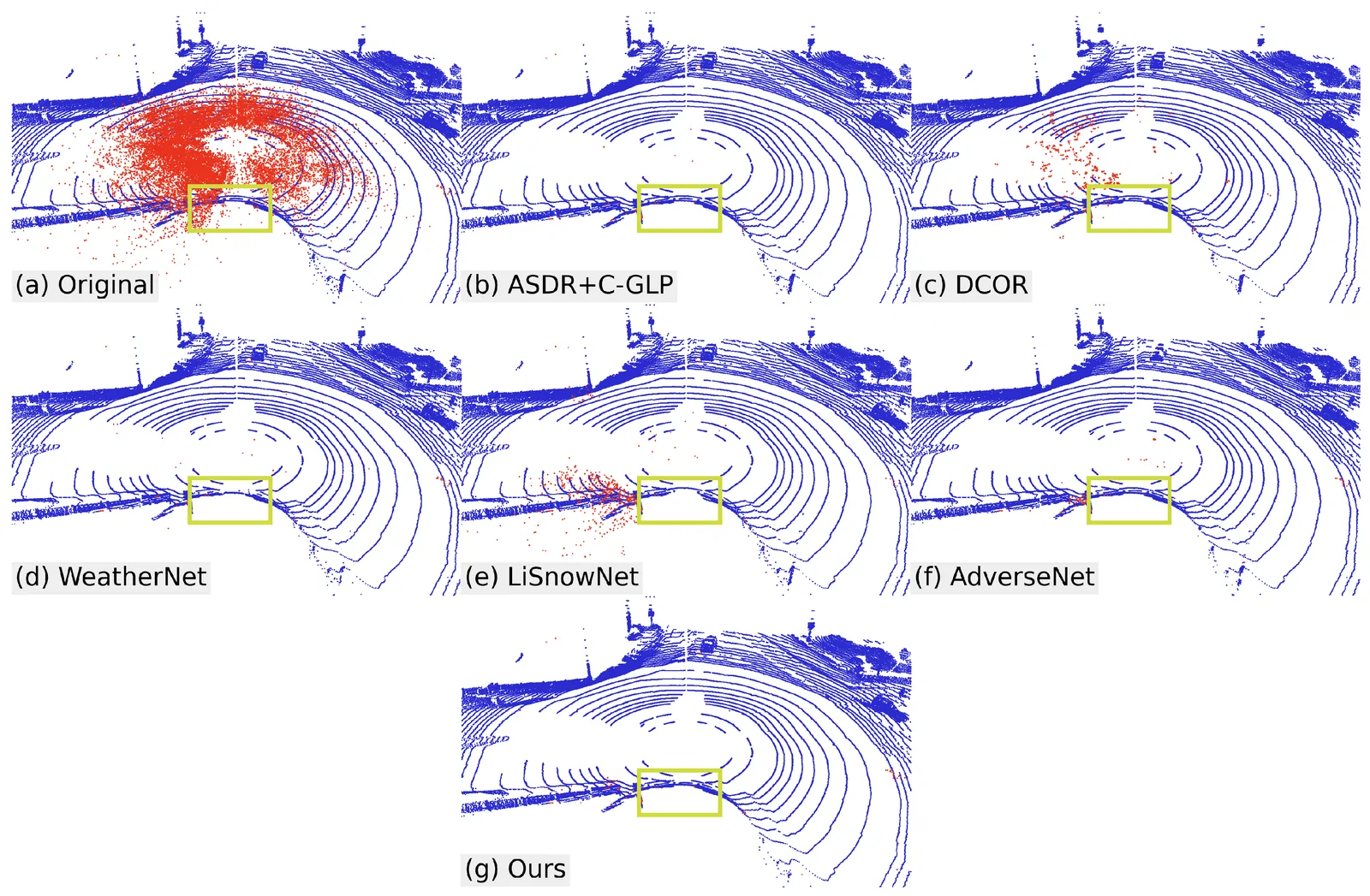
Qualitative comparison of snow-removal results on the WADS dataset. (**a**) Original point cloud. (**b**) ASDR+C-GLP labels. (**c**) DCOR. (**d**) WeatherNet. (**e**) LiSnowNet. (**f**) AdverseNet. (**g**) SnowRemovalNet. Red and blue points indicate snow and non-snow points, respectively, and the yellow boxes highlight the region around the guardrail.

On WADS, as shown in Table 3, the proposed method achieved a Precision of 95.75%, Recall of 98.76%, and F1-score of 97.23%. Unlike CADC, the WADS scene contains snow noise densely distributed around roadside structures such as guardrails. As shown in Figure 6g, the proposed method removed snow noise around the guardrail while stably preserving the guardrail structure. In contrast, LiSnowNet left relatively more residual snow noise around the guardrail.

Table 2 and Table 3 also list the ASDR+C-GLP labels used to train the proposed method (Section 4.2), evaluated with a threshold of 0.5. Their F1-scores of 98.61% on CADC and 98.01% on WADS were higher than the 98.10% and 97.23% of the proposed method. However, ASDR+C-GLP required approximately 0.22 s and 0.93 s per frame on CADC and WADS, respectively, even with a GPU implementation of C-GLP. Therefore, ASDR+C-GLP was used only offline. The proposed method approximated its accuracy within 0.78 percentage points while processing a frame in 17–59 ms.

As listed in Table 2 and Table 3, the F1-scores of AdverseNet were 0.05 and 5.85 percentage points lower than those of the proposed method on CADC and WADS, respectively. WeatherNet also achieved lower F1-scores than the proposed method on both datasets. AdverseNet has 12.7 million parameters, approximately 354 times the 35,925 parameters of the proposed network. Its processing times on CADC and WADS were approximately 8.1 and 2.6 times those of the proposed method, respectively. Thus, the proposed method achieved higher F1-scores with a substantially smaller network and a shorter processing time.

#### 5.2.2. Ablation Study

In the Ours (w/o probabilistic labels) variant, the continuous probabilistic labels in the range of [0,1] were replaced with hard binary labels in {0,1}. Since binary labels do not contain pointwise uncertainty information, the curriculum learning strategy and uncertainty-weighted loss function were not applied in this variant. As shown in Table 2 and Table 3, this variant achieved F1-scores of 94.33% and 94.44% on CADC and WADS, respectively, lower than those of the full model. These results suggest that the probabilistic-label-based framework, including curriculum learning and uncertainty-weighted loss, is effective for ambiguous boundary regions where snow and non-snow points are spatially intermingled.

In the Ours (w/o 2D BEV features) variant, the 2D BEV features and the associated BEV-based gating and FiLM modulation were removed, and the model relied only on DGCNN-based 3D local feature extraction. The F1-scores decreased to 92.58% on CADC and 92.60% on WADS. This performance degradation suggests that relying solely on 3D local geometric features is less effective for capturing the global spatial distribution of snow noise. In contrast, the full model achieved the highest F1-scores among the three variants. Overall, these results suggest that both the probabilistic supervision and the BEV-guided 3D point feature learning contributed to the snow-noise removal performance.

The BEV features are sampled with nearest-neighbor grid_sample from the 75×75 feature map, whose cells are approximately 0.8 m on a side. Therefore, all points in the same feature-map cell receive identical BEV features regardless of their heights. In the test frames, 37.1% of the points within the BEV map on CADC and 30.3% on WADS were height-aliased. Each shared its cell with a point of the other class whose height differed by at least 0.5 m. In an additional experiment, the maximum and minimum heights in each 0.1 m cell were added to the BEV map as two channels. The mean F1-scores of five runs with 30 frames were 97.52% on CADC and 96.22% on WADS. As reported in Section 5.5, the corresponding values with the two-channel BEV map were 97.35% and 95.29%. The differences were not statistically significant according to Welch’s *t*-test, with p=0.77 and 0.35, respectively. Therefore, the two-channel BEV map was retained. The absence of a significant difference is consistent with the 3D branch providing the height information through the relative positions $\Delta p_{ij}$ in (8) and (9).

#### 5.2.3. Qualitative Results on Boreas

**Figure 7:**
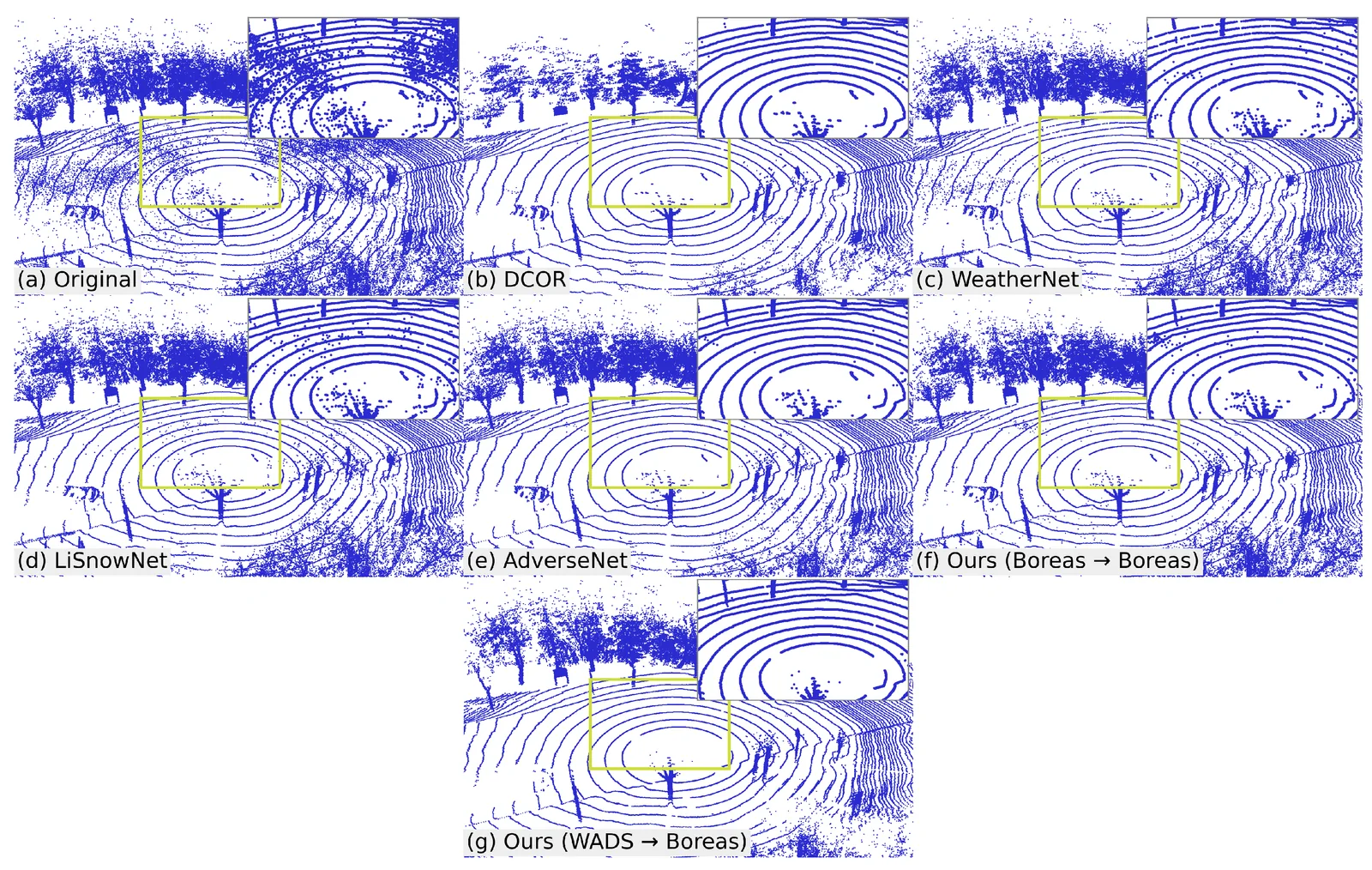
Qualitative comparison of snow-removal results on the Boreas dataset. (**a**) Original point cloud. (**b**) DCOR. (**c**) WeatherNet. (**d**) LiSnowNet. (**e**) AdverseNet. (**f**) SnowRemovalNet trained and tested on Boreas. (**g**) SnowRemovalNet trained on WADS and tested on Boreas. The yellow box in each panel marks the region around the sensor, and the inset at the upper right shows an enlarged view of this region.

The 128-channel Boreas dataset has a relatively high point density among the experimental datasets. Its snow noise and real-world structures are densely mixed, making it difficult to obtain reliable pointwise evaluation annotations. Therefore, a qualitative analysis was conducted. Because Boreas provides no pointwise snow labels, DCOR was applied with its published parameters, and WeatherNet and AdverseNet with the models trained on WADS. Figure 7 shows a trade-off between removing the snow noise completely and preserving the surrounding structures. DCOR removed the snow noise around the sensor almost completely, but it also removed a large portion of the nearby trees and the distant ground (Figure 7b). WeatherNet, LiSnowNet, and AdverseNet left more residual snow points while still removing part of the trees (Figure 7c–e). The two variants of the proposed method cover both sides of this trade-off. The model trained on Boreas preserved the nearby trees and the vehicle with a few residual snow points (Figure 7f). The model trained on WADS left the fewest residual snow points among the learning-based methods, although part of the tree points were also removed (Figure 7g). In addition, the average processing time of 95 ms per frame demonstrated applicability to real-time operation.

### 5.3. Cross-Dataset Generalization

In this section, the model trained on WADS was applied to CADC and Boreas without retraining to assess cross-dataset generalization under different sensor configurations.

Applying the WADS-trained model to the CADC dataset resulted in a Precision of 94.82%, Recall of 97.93%, and F1-score of 96.35%. As shown in Figure 5h, the scattered snow noise was effectively removed while preserving most of the surrounding terrain structures.

As shown in Figure 7g, the model trained on the 64-channel WADS dataset removed most snow noise across the field of view of the 128-channel Boreas scans. A quantitative evaluation through a downstream odometry task is presented in Section 5.4.2. These results suggest that the model captures generalizable snow-noise characteristics rather than overfitting to dataset-specific characteristics.

### 5.4. Downstream Task Evaluation

The effect of de-snowing on downstream tasks was evaluated through semantic segmentation and LiDAR odometry.

#### 5.4.1. Semantic Segmentation

Semantic segmentation was evaluated on the WADS dataset, which provides pointwise semantic labels. The training/test split followed the LiSnowNet study [15], excluding one training sequence whose scans duplicate those of a test sequence. A sparse 3D U-Net [26] was trained after the falling-snow points were removed from the training scans according to their labels. The network therefore does not recognize falling snow, as in perception networks trained with standard semantic classes. Each test scan was segmented before and after de-snowing by the proposed method, and the removed points were counted as misclassified for their labeled class. The intersection over union (IoU) was computed for eight classes and averaged into the mean IoU (mIoU).

**Figure 8:**
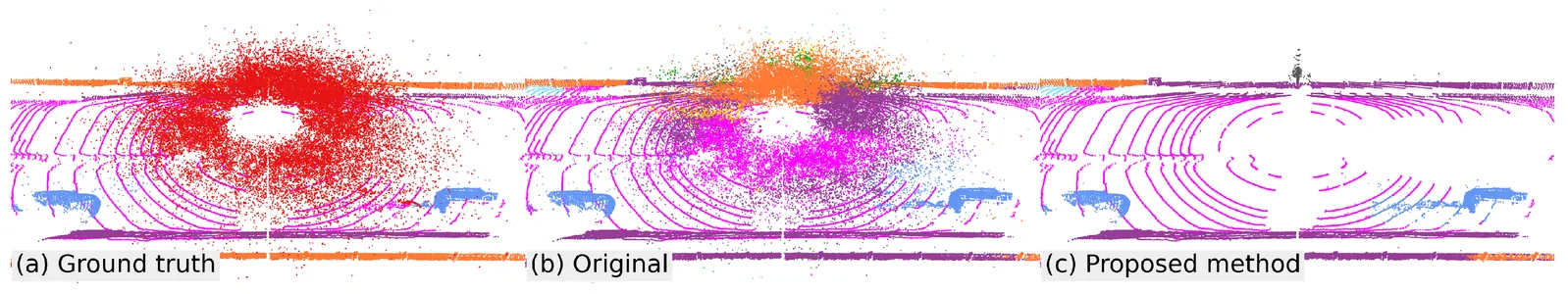
Semantic-segmentation results on a WADS test scan (points within 30 m). (**a**) Ground-truth labels. (**b**) Segmentation of the original point cloud. (**c**) Segmentation of the point cloud de-snowed by the proposed method. The red points in (**a**) indicate falling snow. The other colors denote vehicle (blue), road (magenta), ground (dark purple), building (yellow), structure (orange), vegetation (green), pole (dark gray), and accumulated snow (light blue).

As listed in Table 4, de-snowing with the proposed method increased the mIoU from 44.90% to 48.40%. Within 10 m, the mIoU increased from 31.48% to 48.79%, and the vehicle IoU increased from 33.72% to 74.08%. As shown in Figure 8b, the dense falling-snow points in this range were otherwise segmented as object classes such as road and structure. In contrast, the IoU of the building and accumulated-snow classes decreased by 3.6 and 5.5 percentage points, because part of these points were removed as snow, as observed in Section 5.6. These results indicate that de-snowing with the proposed method benefits semantic segmentation, particularly near the sensor, where snow noise is concentrated.

#### 5.4.2. LiDAR Odometry

The de-snowing results on Boreas were evaluated quantitatively through LiDAR odometry. The test sequence was recorded during heavy snowfall and contains 13,624 scans over a 7.9 km route, with ground-truth poses from a post-processed GNSS/INS solution [25]. Each scan was de-snowed by the model trained on WADS without retraining. Therefore, this experiment also constitutes a quantitative cross-dataset evaluation. The raw scans (Raw) were used as the reference input.

Odometry was estimated by scan-to-model registration with voxelized generalized iterative closest point (VGICP) [27] implemented in small_gicp [28]. Since identical settings were used for both inputs, the differences in accuracy are attributable to the de-snowing step. The relative pose error (RPE) followed the KITTI odometry protocol [29]. The absolute trajectory error (ATE) was computed on 94 sub-segments of 4323 frames starting every 100 frames, each covering 1.9–3.4 km, as the root-mean-square position error after a rigid alignment [30], and averaged over the sub-segments.

As shown in Table 5, de-snowing with the proposed method reduced the translational RPE over the full sequence from 0.645% to 0.614%, and by 3.5–6.4% at every segment length from 100 to 800 m. The rotational RPE remained at 0.150–0.151°/100 m. The mean ATE decreased from 3.70 m to 3.60 m, with decreases in 83 of the 94 sub-segments. These results suggest that de-snowing with the proposed method benefits VGICP-based scan registration.

### 5.5. Training Data Efficiency

To examine whether the 30 frames per dataset used in the preceding experiments are sufficient, the model was additionally trained with 100 and 300 frames per dataset. The 100- and 300-frame sets were constructed by adding frames selected at uniform intervals to the 30-frame set. Frames within 10 frames of the test frames were excluded, so that scenes nearly identical to the test frames were kept out of training. All other settings followed Table 1. For each set, the model was trained five times with different random seeds. Table 6 lists the mean and standard deviation of the results on the test frames.

As shown in Table 6, 30 training frames were sufficient for the proposed method. On CADC, the difference of 0.25 percentage points between the 30-frame (97.35%) and 300-frame (97.60%) mean F1-scores was smaller than the standard deviation over the seeds. On WADS, the 30-frame mean F1-score was 95.29%, compared with 97.90% for the best setting with 100 frames. Thus, with one-tenth of the training frames, the proposed method achieved 99.7% and 98.5% of the 300-frame mean F1-scores on CADC and WADS, respectively, in approximately 9% of the training time.

### 5.6. Performance Under Different Snowfall Intensities

To examine the performance under different snowfall intensities, WADS frames were grouped by their snow-point fraction computed from the pointwise labels. Every tenth frame was sampled from the WADS frames not used for training or testing, resulting in 170 frames. Frames with a snow-point fraction of less than 5%, 5–10%, and 10% or more were grouped into light, moderate, and heavy snowfall, respectively. The frame with the median snow-point fraction of each group was evaluated against its pointwise ground truth with the same protocol as in Table 3.

**Figure 9:**
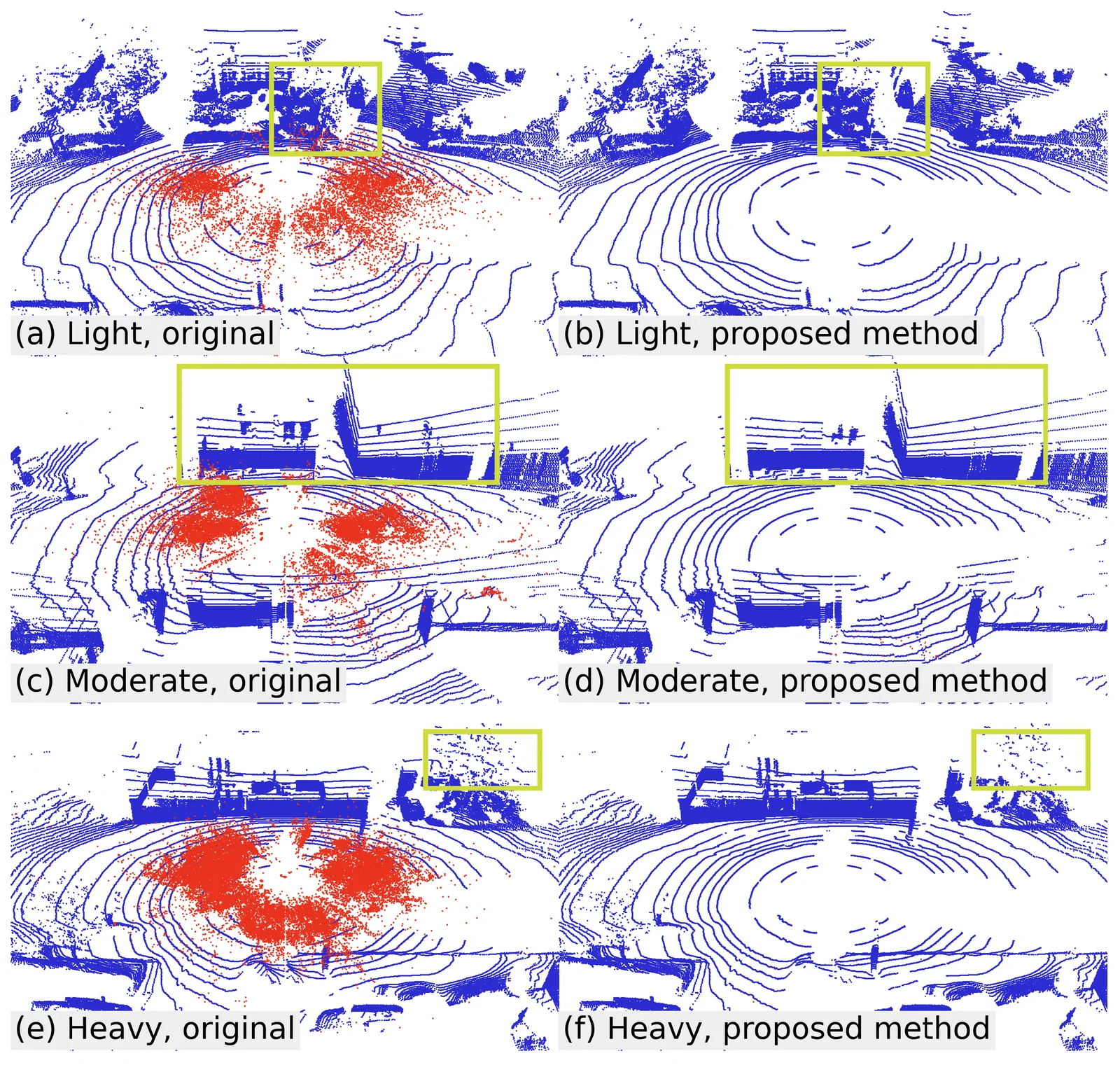
Snow-removal results of the proposed method on the WADS frames with different snowfall intensities. (**a**) Light snowfall, original point cloud. (**b**) Light snowfall, points kept by the proposed method. (**c**) Moderate snowfall, original point cloud. (**d**) Moderate snowfall, points kept by the proposed method. (**e**) Heavy snowfall, original point cloud. (**f**) Heavy snowfall, points kept by the proposed method. Red and blue points indicate snow and non-snow points, respectively, and the yellow boxes mark the regions where tree foliage (light and heavy snowfall) and building points (moderate snowfall) were removed.

As listed in Table 7, the Recall of the proposed method remained above 99.7% in all three frames. In contrast, the Precision increased from 68.79% under light snowfall to 74.98% and 84.34% under moderate and heavy snowfall, respectively, and the F1-score from 81.47% to 85.61% and 91.48%. The light and heavy frames contain trees near the sensor, and their foliage points are as sparse as snow points. As shown in the yellow boxes in Figure 9b,f, the proposed method removed part of these foliage points as snow, which lowered the Precision. The false removals in the moderate frame were mainly building points, as shown in the yellow box in Figure 9d. Because snow points accounted for only 4.54% of the light frame, these false removals lowered the Precision more strongly than under heavier snowfall. The test frame of Table 3 contains few such sparse structures near the sensor. The lower F1-scores of these frames therefore result mainly from the false removal of the foliage and building points. These results indicate that the proposed method detected snow points consistently across snowfall intensities. They also indicate that the main failure mode of the proposed method is the removal of sparse non-snow points that resemble snow, such as tree foliage.

## 6. Conclusions

In this study, we proposed a deep learning-based LiDAR de-snowing framework that incorporates BEV-based 2D contextual information into 3D point feature learning. Experimental results demonstrated that integrating BEV representations, which capture the global distribution of snow noise, with 3D point features improved snow-noise removal while preserving object structures.

To account for the uncertainty of ambiguous points, probabilistic labels were employed instead of conventional binary labels and further utilized in curriculum learning and the uncertainty-weighted loss design. The network was trained with only 30 frames per dataset, requiring approximately 9% of the 300-frame training time while achieving 98.5–99.7% of its F1-score.

In experiments using the CADC, WADS, and Boreas datasets, the proposed method reliably removed snow noise under diverse sensor configurations and point density conditions while satisfying real-time processing requirements. Furthermore, the model trained on WADS exhibited promising cross-dataset generalization capability when applied to the CADC and Boreas datasets without retraining. In a downstream LiDAR odometry evaluation on a Boreas sequence recorded during heavy snowfall, the WADS-trained model also reduced the translational relative pose error from 0.645% to 0.614%. In a downstream semantic-segmentation evaluation on WADS, de-snowing by the proposed method increased the mIoU of a segmentation network trained without a falling-snow class from 44.90% to 48.40%.

## Figures and Tables

**Table 1:** Implementation details of SnowRemovalNet. Values spanning both dataset columns are shared by the CADC and WADS models.

Category	Item	CADC	WADS
BEV Encoder	BEV grid: XY extent, cell size	±30 m, 0.1 m	
	BEV map size (2×H×W)	2×600×600 (intensity, density)	
	Channels: input, $C_{1}$, $C_{2}$, $C_{3}$, $C_{out}=C_{b}$	2→16→32→64→16	
	Feature map size; pointwise sampling	75×75; nearest-neighbor grid_sample	
Point branch	Point feature dimension	64	
	*k*, Edge Conv Block 1	16 (KNN in XYZ space)	
	*k*, Edge Conv Block 2	12 (KNN on a fixed 16-D projection of the features)	
	Prediction Head	64→64→1	
	Total parameters	35,925	
Training	Frames (training/validation)	30 (21/9)	
	Points per sample ($N_{s}$)	1024	2048
	Batch size	16	8
	Learning rate (constant)	0.01	0.001
	Optimizer; weight decay	Adam; $10^{-4}$	
	Epochs (*E*)	100	
	Curriculum stages, as in (4)	Epochs 1–34, 35–67, 68–100	
	Data augmentation	None	
	Checkpoint selection	Lowest validation loss in stage 3	

**Table 2:** Performance comparison of snow-removal methods on CADC.

Category	Method	Learning Time (s, ↓)	Proc. Time (ms, ↓)	Precision (%, ↑)	Recall (%, ↑)	F1-Score (%, ↑)
Traditional statistics- based	DSOR [1]	–	42.04	91.21	97.43	94.22
	DROR [2]	–	31.25	34.55	99.26	51.26
	DDIOR [3]	–	33.80	40.64	99.22	57.66
	AGDOR [4]	–	16.87	42.01	98.92	58.97
	LIOR [5]	–	7.01	94.72	96.04	95.37
	Adaptive Two-Stage Filter [7]	–	4.10	69.40	93.83	79.79
	Neighbor-Intensity-Based [8]	–	48.24	75.13	45.10	56.36
	DCOR [12]	–	17.30	80.34	93.86	86.57
Deep learning	WeatherNet [13]	127	22	83.54	98.21	90.28
	LiSnowNet [15]	52	10	80.37	93.04	86.24
	AdverseNet [14]	1622	138	97.98	98.12	98.05
	Ours (w/o probabilistic labels)	45	12	92.75	95.96	94.33
	Ours (w/o 2D BEV features)	44	20	90.12	95.18	92.58
	Ours	48	17	98.09	98.10	98.10
Offline labels	ASDR+C-GLP [11]	–	217	98.94	98.28	98.61

Note: ↑/↓ indicates “higher/lower is better”.

**Table 3:** Performance comparison of snow-removal methods on WADS.

Category	Method	Learning Time (s, ↓)	Proc. Time (ms, ↓)	Precision (%, ↑)	Recall (%, ↑)	F1-Score (%, ↑)
Traditional statistics- based	DSOR [1]	–	181.24	88.23	98.12	92.91
	DROR [2]	–	173.13	45.12	96.99	61.59
	DDIOR [3]	–	125.41	34.12	98.21	50.64
	AGDOR [4]	–	70.75	45.81	98.89	62.61
	LIOR [5]	–	42.38	88.55	99.14	93.55
	Adaptive Two-Stage Filter [7]	–	7.39	75.75	99.11	85.87
	Neighbor-Intensity-Based [8]	–	165.36	85.16	99.03	91.57
	DCOR [12]	–	81.21	82.57	96.03	88.79
Deep learning	WeatherNet [13]	270	55	70.68	99.63	82.69
	LiSnowNet [15]	622	16	75.15	96.07	84.33
	AdverseNet [14]	1823	153	84.51	99.47	91.38
	Ours (w/o probabilistic labels)	68	49	94.12	94.76	94.44
	Ours (w/o 2D BEV features)	65	68	89.21	96.26	92.60
	Ours	72	59	95.75	98.76	97.23
Offline labels	ASDR+C-GLP [11]	–	927	96.37	99.70	98.01

Note: ↑/↓ indicates “higher/lower is better”.

**Table 4:** Semantic-segmentation performance on the WADS test sequences before and after de-snowing by the proposed method.

	IoU (%, ↑)							
Input	Vehicle	Road	Ground	Building	Structure	Vegetation	Pole	mIoU (%, ↑)
Raw	63.09	49.43	11.04	**77.82**	11.28	**45.89**	43.53	44.90
Ours	**68.85**	**66.95**	**17.11**	74.21	**15.68**	45.61	**47.17**	**48.40**

Note: ↑ indicates “higher is better”. The mIoU is averaged over eight classes, including the accumulated-snow class (57.08 for Raw and 51.59 for Ours), which is omitted from the table.

**Table 5:** VGICP odometry accuracy on the Boreas test sequence with and without de-snowing by the proposed method.

		Full Sequence (7.9 km)		Sub-Segments of 4323 Frames (94)
Input	Points Kept (%)	RPE t (%, ↓)	RPE r (°/100 m, ↓)	Mean ATE (m, ↓)
Raw	100.0	0.645	0.151	3.70
Ours	77.4	**0.614**	**0.150**	**3.60**

Note: ↓ indicates “lower is better”.

**Table 6:** Effect of the number of training frames on the proposed method. The training time is relative to the 30-frame setting.

Dataset	Frames (Training/Validation)	Parameter Updates	Relative Training Time	Precision (%, ↑)	Recall (%, ↑)	F1-Score (%, ↑)
CADC	30 (21/9)	100	×1.0	97.34 ± 2.43	97.42 ± 0.98	97.35 ± 1.00
	100 (70/30)	400	×3.7	99.37 ± 0.50	94.20 ± 1.70	96.71 ± 0.74
	300 (210/90)	1300	×11.2	99.43 ± 0.29	95.85 ± 0.95	97.60 ± 0.40
WADS	30 (21/9)	200	×1.0	91.32 ± 3.24	99.69 ± 0.05	95.29 ± 1.73
	100 (70/30)	800	×3.7	99.79 ± 0.06	96.10 ± 1.87	97.90 ± 0.96
	300 (210/90)	2600	×11.8	99.85 ± 0.07	93.95 ± 3.72	96.77 ± 2.00

Note: ↑ indicates “higher is better”.

**Table 7:** Snow-removal performance of the proposed method on WADS frames with different snowfall intensities.

Snowfall	Points	Snow Points (%)	Precision (%, ↑)	Recall (%, ↑)	F1-Score (%, ↑)
Light	210,878	4.54	68.79	99.90	81.47
Moderate	210,274	7.09	74.98	99.74	85.61
Heavy	211,788	14.71	84.34	99.94	91.48

Note: ↑ indicates “higher is better”.

## Data Availability

All datasets analyzed in this study are publicly available third-party datasets. The Canadian Adverse Driving Conditions (CADC) dataset [23] is available at http://cadcd.uwaterloo.ca/ (accessed on 15 October 2026). The Winter Adverse Driving dataSet (WADS) [24] is available at https://digitalcommons.mtu.edu/wads/ (accessed on 15 October 2026). The Boreas dataset [25] is available at https://www.boreas.utias.utoronto.ca/ (accessed on 15 October 2026).
